# Mapping pre-hospital research presented at UK conferences between 2010 and 2023: a bibliometric study

**DOI:** 10.29045/14784726.2026.3.10.4.18

**Published:** 2026-03-01

**Authors:** Graham McClelland, Dan Haworth, Karl Charlton, Lee Thompson, Tracy Finch, Julia Williams

**Affiliations:** Northumbria University ORCID iD: https://orcid.org/0000-0002-4502-5821; North East Ambulance Service NHS Foundation Trust ORCID iD: https://orcid.org/0000-0003-0334-3300; North East Ambulance Service NHS Foundation Trust ORCID iD: https://orcid.org/0000-0002-9601-1083; North East Ambulance Service NHS Foundation Trust ORCID iD: https://orcid.org/0000-0002-0820-1662; Northumbria University ORCID id: https://orcid.org/0000-0001-8647-735X; University of Hertfordshire ORCID iD: https://orcid.org/0000-0003-0796-5465

**Keywords:** conference, mapping, paramedic

## Abstract

**Introduction:**

The growth of the paramedic profession over recent years is reflected in the growing body of publications by paramedics or related to paramedics, ambulance services and pre-hospital care. Publications are not the only method by which new knowledge can be disseminated, and conferences represent another method of dissemination. Conference presentations may or may not be published, so studying these presents a different perspective on topics of interest and research happening within the profession. This study set out to report on material presented at large conferences relevant to UK paramedics between 2010 and 2023.

**Methods:**

The project comprised a bibliometric study describing presentations from UK conferences relevant to paramedics between 2010 and 2023. Conferences relevant to paramedic practice were selected by the study team based on pre-determined criteria. Standardised forms were used to extract data on presentations and presenters. Data are presented descriptively.

**Results:**

Six large conferences (999 EMS Research Forum, Ambulance Leadership Forum, Faculty of Pre-Hospital Care Conference, College of Paramedics National Conference, Research Conference and Student Conference) were selected, and data from 43 individual conferences were collected, representing 70% of the potential conferences during this time frame. The data include 690 presentations given by 551 individual presenters. Paramedics were the most common professional group presenting. The London Ambulance Service, North East Ambulance Service and University of Sheffield were the most common institutions represented. The most common topics under discussion were policy and practice, research and trauma. The most common methodologies were qualitative.

**Conclusion:**

This study provides an overview of research presented at paramedic, ambulance service and pre-hospital conferences. A wide range of research was presented at the selected conferences by many individuals. A wide range of topics feature in the data, but high-impact, low-frequency clinical conditions, such as cardiac arrest and major trauma, feature highly.

## Introduction

Ambulance services are at the heart of the urgent and emergency care system in England (NHS England, 2025a), and the care delivered by paramedics covers a wide spectrum of patients and conditions. Pre-hospital care has rapidly evolved, as paramedic practice advances and paramedics move into new roles to help meet changing patient demand. At the same time, there has been an exponential growth in the volume of pre-hospital research in recent years (Armstrong et al., 2021; Maurin Söderholm et al., 2019), which has coincided with the increasing professionalisation of paramedics and the move to university-based education. The growth in this area can be seen in the emergence of paramedicine-focused journals and conferences both in the United Kingdom (UK) and internationally.

Recent publications have described the development of pre-hospital research using bibliometric data on journal publications (Beovich et al., 2021; Goldberg et al., 2023; Xu et al., 2021). These studies describe a growing body of research based on publications in peer-reviewed journals from around the world. Journal publications are not the only method by which research is communicated or measured, and conferences are a key part of the research landscape within a discipline, profession or field. Conferences are often where research findings are first presented and so may represent the first stage of dissemination. In addition, research presented at conferences may not be published, or there may be a delay between presentation and publication, so conferences offer unique opportunities to disseminate and highlight research (Mcglynn & Mycock, 2011).

This project used a bibliometric approach to describe presentations from UK pre-hospital conferences to map the field and explore how presented research compares with previously identified priorities and the bibliometric data on publications. This should help evidence the growing research culture in paramedicine, guide future research and potentially contribute towards an updated priority-setting exercise.

### Aim and objectives

The aim of this project was to describe presentations from UK conferences relevant to paramedic practice between 2010 and 2023.

The objectives of the project were to:

1. Map out and describe research presented at UK conferences that was targeted at paramedics and pre-hospital clinicians between 2010 and 2023.
2. Compare presented research to identified priorities and bibliometric data on paramedicine publications.
3. Compare presented research to the nature of calls/presentations from within the population served by pre-hospital providers in the UK.

## Methods

A bibliometric study describing presentations from UK conferences relevant to paramedics was conducted between January and June 2024. Conferences relevant to paramedic practice were selected by the study team, based on the following sets of criteria.

Inclusion criteria:

- Held solely in the UK or Great Britain and Northern Ireland.
- Held regularly between 2010 and 2023.
- Focused on care delivered in the pre-hospital setting by ambulance clinicians.
- Target audience includes ambulance clinicians.

Exclusion criteria:

- Single disease-/condition-/locality-specific conferences.
- Military medicine conferences.
- Helicopter Emergency Medical Service (HEMS)-focused conferences.

Based on these criteria, six conferences were selected for inclusion in the study: 999 EMS Research Forum (999 EMS), Ambulance Leadership Forum (ALF), Faculty of Pre-Hospital Care (FPHC) Conference, College of Paramedics (CoP) National Conference, Research Conference and Student Conference.

Three members of the study team (DH, KC, LT) extracted data on oral presentations from the selected conferences using publicly available conference programmes found online and contact with conference organisers in early 2024. Oral presentations will have included both invited speakers and speakers selected via abstract submissions.

Where available, data were extracted on: title; presenter(s) name, sex, affiliation and profession; disease/condition/topic; format; methodology; geographical area; and funder. Where missing data could be found via internet searches or personal contacts these were completed. Data were entered into a standardised Excel spreadsheet, then collated and combined into descriptive categories for reporting by GM.

The BIBLIO checklist has been used to support the writing of this article (Montazeri et al., 2023).

## Results

Table 1 summarises the conferences from which data were collected.

**Table 1.** Conferences and whether data were available for each year.

	999 EMS Research Forum	Ambulance Leadership Forum	Faculty of Pre-Hospital Care	CoP National	CoP Research	CoP Student
**2010**	Yes	Unknown	Unknown	No conf	No conf	No conf
**2011**	Unknown	Unknown	Unknown	No conf	No conf	No conf
**2012**	Unknown	Unknown	Unknown	No conf	No conf	No conf
**2013**	Yes	Yes	Unknown	No conf	No conf	Unknown
**2014**	Yes	Yes	Unknown	Unknown	No conf	Unknown
**2015**	Yes	No conf	Unknown	No conf	Yes	Unknown
**2016**	Yes	Yes	Yes	Yes	No conf	Unknown
**2017**	Yes	Yes	Yes	Yes	No conf	Yes
**2018**	Yes	Yes	Yes	Yes	Yes	Yes
**2019**	Yes	Yes	Unknown	Yes	Yes	Yes
**2020**	Yes	No Conf	Unknown	No conf	Yes	Yes
**2021**	Yes	Yes	No conf	Yes	Yes	Yes
**2022**	Yes	Yes	No conf	Yes	No conf	No conf
**2023**	Yes	Yes	Yes	Yes	No conf	Yes

CoP = College of Paramedics; green ‘Yes’ = data collected; yellow ‘No conf’ = confirmed no conference; no colour = unknown.

Assuming each conference was held once each year under normal circumstances, there was a potential maximum of 84 conferences. Data were collected from 43 individual conferences; 23 conferences were confirmed not to have happened and the occurrence of 18 conferences was unknown. Therefore, the data collected represent 70% of the conferences that potentially happened.

The data collected included 690 presentations, given by 551 individual presenters. Most presentations (n = 614, 89%) were given by a single individual. Only 8% (n = 52) of presentations were given by multiple presenters (mean 2.5, SD 0.8, maximum 5). In 3% (n = 23) of presentations the presenter(s) were unrecorded or were collectives. The sex of the presenter was documented where known (n = 743), showing that 61% of presenters were male.

The individuals presenting most frequently within this data were:

- Graham McClelland (n = 13, 2%)
- Julia Williams (n = 12, 2%)
- Kim Kirby (n = 8, 1%)
- Peter Eaton Williams (n = 6, 1%)
- Janette Turner, Joshua (Josh) Miller, Aloysius Niroshan (Niro) Siriwardena, Richard Pilbery, Sam Thompson (n = 5, 1%)

The individuals presenting most frequently by conference were:

- 999 EMS (n = 162)
  - Julia Williams (n = 6, 4%)
  - Graham McClelland, Imogen Gunson, Niro Siriwardena (n = 4, 2%)
  - Edward Duncan, Kim Kirby (n = 3, 2%)
- ALF (n = 145)
  - Janette Turner, Kerry Gulliver (n = 3, 2%)
  - Chris Lucas, Euros Evans, Karina Catley, Keith Willet, Nigel Edwards, Paresh Wankhade, Peter Eaton Williams, Ruth Crabtree, Steven Scholes, Tim Edwards, Yvonne Coghill (n = 2, 1%)
- FPHC (n= 82)
  - Andy Smith, Esther Murray, Hans Morten Lossius, Richard Lyon, Stacey Webster, Tim Nutbeam (n = 2, 2%)
- CoP National (n = 206)
  - Graham McClelland (n = 5, 2%)
  - Andy Collen, Georgette Eaton, Julia Williams (n = 3, 1%)
  - Kim Kirby, Paul Gowens, Sam Thompson (n = 2, 1%)
- CoP Research (n = 105)
  - Graham McClelland (n = 4, 4%)
  - Julia Williams (n = 3, 3%)
- CoP Student (n = 45)
  - Andy Thomas (n = 4, 9%)
  - Alex Ulrich, James Yates, Sam Thompson (n = 2, 4%)

The institutions (n = 474) most frequently represented were London Ambulance Service (n = 17, 4%), North East Ambulance Service and University of Sheffield (both n = 16, 3%), then North West Ambulance Service, South East Coast Ambulance Service, Swansea University, Welsh Ambulance Service and Yorkshire Ambulance Service (all n = 15, 3%).

Presenter professions (n = 344) were grouped into similar categories due to the variety of job titles reported. The authors used the most obvious profession reported in the job title. The most common professional groups were paramedic (n = 115, 33%), manager/chief/director (n = 90, 26%), academic (n = 64, 19%), medical doctor (n = 60, 17%) and other clinician (n = 11, 3%).

The foci of the presentations (n = 690) were grouped based on disease, condition or topic into 131 categories. The most common groupings were policy and practice (n = 42, 6%), research (including methods, networks, evidence-based medicine and other associated content) (n = 39, 6%), trauma (n = 39, 6%), out-of-hospital cardiac arrest (n = 31, 4%), education (n = 25, 4%), equality, diversity and inclusivity (n = 22, 3%), COVID-19 (n = 21, 3%), mental health (n = 17, 2%), leadership (n = 16, 2%) and paediatrics (n = 16, 2%).

The self-reported methodology used was recorded where described (n = 118). The most common methodologies or approaches were qualitative (n = 30, 25%), observational (n = 21, 18%), literature review (n = 15, 13%), randomised study (n = 12, 10%), mixed methods (n = 6, 5%), pilot/feasibility (n = 5, 4%) and ethnographic (n = 2, 2%).

Where geographical area was described, this was recorded (n = 151). Locations commonly mentioned were outside the UK (n = 64, 42%) or across the UK (n = 35, 23%). Specific named locations included England (n = 8, 5%), the North East (n = 10, 7%) and London (n = 6, 4%).

Presentation format was overwhelmingly oral presentation (n = 686, 99%), with panel discussion (n = 3) and a single debate accounting for the other presentations. Co-authors and funders were very rarely reported (3% and 1%), so are not described further here.

## Discussion

This project described presentations from six large, regular conferences focused on paramedic practice and pre-hospital and emergency care between 2010 and 2023. A broad range of presentation topics were identified and delivered by a multitude of speakers from a range of professional backgrounds. This study presents a unique snapshot of research conducted by, and topics of interest to, UK paramedics, based on conference presentations.

The time frame described included the COVID-19 pandemic, and many conferences were either paused or were delivered online during this period. Despite only being relevant in the final years of the time covered, COVID-19 still accounted for 3% of presentations. COVID-19 undoubtedly impacted pre-hospital research, with non-COVID-19 studies being paused, research staff being redeployed and staff who may have started research projects or careers unable to do so. The longer-term impact of this on paramedic research is yet unclear.

While a wide range of ambulance services were identified as frequently presenting, a smaller number of universities featured regularly. The universities identified in the data (Sheffield and Swansea) are both well known for their work in this area and have leaders in the field of pre-hospital and emergency research. There are a small number of active researchers focused on paramedic practice and pre-hospital care, with limited options to present their work to large paramedic audiences, so it is unsurprising to see the same individuals from the pre-hospital research community featuring heavily in the selected conferences.

The most common clinical foci for presentations (trauma and cardiac arrest) are notable, as they comprise a small percentage of the patient population but receive a large amount of research and interest as demonstrated here. These clinical conditions are often high-acuity but low-occurrence conditions. The prominence of these types of conditions, as opposed to conditions with lower acuity but which are seen with higher frequency, such as frailty and mental health, are worthy of further consideration.

It is difficult to concisely describe the population served by UK pre-hospital care providers, although some literature provides an indication of what type of calls make up this population. Henderson et al. (2019) broadly described the population seen based on a small sample of patient records from South West England and identified trauma as the most frequent incident code, with other medical and gastrointestinal complaints coming second and third. Data from NHS England suggest that resuscitation (as a marker of cardiac arrest calls) was attempted in <0.5% of ambulance service calls between 2018 and 2024 (NHS England, 2025b). Data from the US suggest that OHCA accounts for around 1% of calls based on emergency medical services (EMS) provider impression (NEMSIS, 2025). Major trauma is estimated to account for 0.3% of ambulance service calls (Maddock et al., 2020).

Pre-hospital management of cardiac arrest is a key skill ambulance clinicians learn and practice. Cardiac arrest is perhaps a frequently presented topic due to its perceived importance in paramedic practice, large studies that have challenged practice during the time covered (AIRWAYS2 (Benger et al., 2022), PARAMEDIC2 (Perkins et al., 2018)) and ongoing controversies and discussions around practice. Other broad areas that were commonly found in the data include policy and practice, research and leadership, which may reflect the specific areas of interest of the selected conferences.

It was notable that among the research methodologies identified in the data, there was a high number of qualitative studies, making up 25% of the reported methodologies, and there was a low number of randomised controlled trials. This may reflect the growing development of paramedic research over the time frame described, as well as the challenges of undertaking clinical studies such as randomised controlled trials in the pre-hospital setting.

Research priority setting encompasses any activities that involve stakeholders in identifying, prioritising and reaching consensus on those areas, topics or questions that research needs to address (Tong et al., 2019). Priority-setting exercises are important, as they help to focus future research on important topics and they influence research funding. There have been two large priority-setting exercises in UK pre-hospital care. Snooks et al. (2008) conducted a Delphi exercise to identify high-priority areas for pre-hospital care. In 2010 the Department of Health published ‘Building the evidence base in pre-hospital urgent and emergency care’ (Turner et al., 2010), which identified the main evidence gaps in pre-hospital, urgent and emergency care and summarised the research on 11 key topics.

Since the Snooks and Turner publications, priorities have been identified for other systems, settings and/or groups, such as Ireland, Australia and New Zealand, and UK pre-hospital critical care (Bowles et al., 2024; Harthi et al., 2022; Pap et al., 2024; Ramage et al., 2023; Vloet et al., 2021). Due to the broad grouping of topics in this article, and variations in the terminology used, it is difficult to directly compare the findings of this study to previously identified priorities. A UK pre-hospital research priority-setting exercise is currently under development involving the Royal College of Paramedics, the James Lind Alliance and other stakeholders.

Olaussen et al. (2021) identified the top 100 cited articles in SCOPUS that related to paramedicine. They looked at published articles within an international sample, so there are differences in the sample and the medium, but there are also some similarities to note. They found that 70% of authors were male, which is similar to our findings (61%), and that observational studies were most frequent. However, they found qualitative studies were the least frequent, which is unlike the findings in this mapping project, where qualitative studies were most frequent and may be related to the journals included in SCOPUS. They also reported that out-of-hospital cardiac arrest was the most frequent clinical condition in the top articles, which aligns with cardiac arrest being in the top five topics presented in this article.

Like Olaussen et al., Beovich et al. (2021) conducted a bibliometric analysis of paramedic publications between 2010 and 2019 and identified the top two clusters of topics as cardiac, including cardiac arrest and respiratory problems including airway management and intubation. They also highlighted students and education as the top area of research. This work was focused on American and Australian publications, but the prominence of cardiac arrest is again noteworthy. Goldberg et al. (2023) described trends in EMS publications between 2010 and 2020 and highlighted OHCA and trauma as the most published conditions.

Holland and Dutton (2020) reported a bibliographic analysis of paramedic research literature in AMBER (Ambulance Research Repository) between 2011 and 2019. This highlighted some of the same high-frequency authors/presenters and the range of topics covered by the articles with the highest citation counts and Altmetric scores, although OHCA appeared frequently in both categories. Cavanagh et al. (2023) reviewed paramedic research literature from 2006 to 2019 and described a narrow range of topics, with clinical foci such as OHCA occurring commonly, and limited methods being used, with observational studies being most frequent.

### Limitations

This project has several limitations. There are many conferences of interest to ambulance clinicians due to the breadth of conditions seen in pre-hospital care and the growth of the profession. This project focused on a small number of large regular conferences; however, there are other conferences that could be included if the project was repeated and extended. There were data missing about some conferences, and the use of conference programmes and presentation titles limited the level of detailed information available. Assumptions were made about the sex of presenters based on names, which may be incorrect, although efforts were made to clarify these via internet searches when unclear. Presenter professions and presentation foci were grouped in an unstructured way and with some overlap (e.g. one presenter could be both a paramedic and an academic).

Further work in this area could include conference posters to increase the scope of this project but would rely on conferences publishing posters. The AMBER repository publishes posters including those presented at 999 EMS, but this does not always happen (Dutton et al., 2024). Another area where this work could be developed would be exploring how many conference presentations led to publications. Scherer et al. (2018) found that only 37% of conference abstracts led to publications. Studying this requires a longitudinal approach due to the often-prolonged length of time between results being presented and published.

## Conclusion

In the UK, pre-hospital research presented at the selected conferences appears to be delivered by a large number of presenters, including a small number of active researchers. While many areas of paramedic practice and policy are covered, research related to OHCA and trauma appear frequently in conference agendas despite being infrequently seen in practice. In recent years, areas of practice within these clinical specialities have been the focus of large clinical trials delivered in ambulance services, which have naturally stimulated debate within the paramedic profession, so the dominance of these topics is justified. However, the preoccupation with topics that account for a very small amount of ambulance workload (e.g. OHCA, trauma) may exclude other priority areas, such as mental health. Future work to identify UK pre-hospital research priorities will help guide paramedic practice and research, and will ensure that both meet and address the needs of stakeholders.

## Author contributions

GM devised the project, supported data collection and analysis and drafted and revised the manuscript. DH, KC and LT supported project development, collected data, contributed to data analysis and reviewed the manuscript. TF and JW supported project development, contributed to data analysis and reviewed the manuscript. GM acts as the guarantor for this article.

## Conflict of interest

GM and JW are Associate Editors of the *British Paramedic Journal*.

## Ethics

This project received ethical approval from Northumbria University (ref 6995, 12 March 2024).

## Funding

This project was funded by a small grant from the Northumbria University Internal Funding Scheme 2023/2024.

## Statement of generative AI in scientific writing

The authors did not use a generative artificial intelligence (AI) tool or service to assist with preparation or editing of this work.
